# Metabolic phenotyping of acquired ampicillin resistance using microbial volatiles from *Escherichia coli* cultures

**DOI:** 10.1111/jam.15716

**Published:** 2022-08-02

**Authors:** Breanna Dixon, Waqar M. Ahmed, Abubaker A. Mohamed, Tim Felton, Stephen J. Fowler

**Affiliations:** ^1^ Division of Immunology, Immunity to Infection and Respiratory Medicine, Faculty of Biology, Medicine and Health, School of Biological Sciences University of Manchester Manchester UK; ^2^ Manchester Institute of Biotechnology University of Manchester Manchester UK; ^3^ Department of Materials, Faculty of Science and Engineering University of Manchester Manchester UK; ^4^ NIHR Manchester Biomedical Research Centre Manchester University Hospitals NHS Foundation Trust Manchester UK

**Keywords:** ampicillin, antimicrobial resistance, *Escherichia coli*, gas chromatography‐mass spectrometry, metabolic profiling, volatile organic compounds

## Abstract

**Aims:**

This study sought to assess the volatile organic compound (VOC) profiles of ampicillin‐resistant and ‐susceptible *Escherichia coli* to evaluate whether VOC analysis may be utilized to identify resistant phenotypes.

**Methods and Results:**

An *E. coli* BL21 (DE3) strain and its pET16b plasmid transformed ampicillin‐resistant counterpart were cultured for 6 h in drug‐free, low‐ and high‐concentrations of ampicillin. Headspace analysis was undertaken using thermal desorption‐gas chromatography‐mass spectrometry. Results revealed distinct VOC profiles with ampicillin‐resistant bacteria distinguishable from their susceptible counterparts using as few as six compounds. A minimum of 30 compounds (fold change >2, *p* ≤ 0.05) were differentially expressed between the strains across all set‐ups. Furthermore, three compounds (indole, acetoin and 3‐methyl‐1‐butanol) were observed to be significantly more abundant (fold change >2, *p* ≤ 0.05) in the resistant strain compared to the susceptible strain both in the presence and in the absence of drug stress.

**Conclusions:**

Results indicate that *E. coli* with acquired ampicillin resistance exhibit an altered VOC profile compared to their susceptible counterpart both in the presence and in the absence of antibiotic stress. This suggests that there are fundamental differences between the metabolisms of ampicillin‐resistant and ‐susceptible *E. coli* which may be detected by means of VOC analysis.

**Significance and Impact of the Study:**

Our findings suggest that VOC profiles may be utilized to differentiate between resistant and susceptible bacteria using just six compounds. Consequently, the development of machine‐learning models using VOC signatures shows considerable diagnostic applicability for the rapid and accurate detection of antimicrobial resistance.

## INTRODUCTION

The prevalence of antimicrobial resistance (AMR) is growing at an alarming rate and represents a major global public health concern (Antimicrobial Resistance Collaborators, 2022). Methods routinely employed in clinical practice for the detection of resistance involve long incubation steps of up to 24 h and are based on subjective breakpoint criteria rather than direct detection of resistance markers (The European Committee on Antimicrobial Susceptibility Testing, 2021). In addition to being time‐consuming, the disadvantage of these methods is that resistance mechanisms may go undetected if the susceptibility criteria are not met, with potentially negative implications for both patient outcomes and the spread of drug‐resistant strains (Kidd et al., 2020; Wiskirchen et al., 2014). There is an urgent need to develop cost‐effective methods for the detection of AMR which have a rapid turnaround time and high accuracy, for implementation into routine clinical diagnostics.

*Escherichia coli* are a major cause of nosocomial infections including ventilator‐associated pneumonia, hospital‐acquired urinary tract infections and surgical site infections (Khan et al., 2015; Trubiano & Padiglione, 2015). According to the Centers for Disease Control and Prevention National Healthcare Safety Network between 2015 and 2017, *E. coli* was the most frequently reported pathogen across all hospital‐acquired infections, accounting for 18% of all infections (Weiner‐Lastinger et al., 2020). The consequence of multidrug resistance within these organisms poses significant therapeutic challenges and is of particular concern.

AmpC β‐lactamase production may arise through the inducible expression of the chromosomal *ampC* gene via mutations of the promoter or regulatory genes, including *ampR*, or through transmissible plasmids harbouring *ampC* genes and enabling high‐level production of AmpC β‐lactamases (Caroff et al., 1999). AmpC production is associated with resistance to a broad range of antibiotics, including third‐generation cephalosporins and reduced inhibition by commonly used β‐lactamase inhibitors such as clavulanic acid and tazobactam (Belley et al., 2021). Furthermore, AmpC β‐lactamases may confer resistance to carbapenems when coupled with porin mutations which reduce outer membrane permeability (Hamzaoui et al., 2018).

Bacteria display species‐specific profiles of volatile organic compounds (VOCs) and these may be used as markers of pathogen infection in some disease states (Ahmed et al., 2017; Bos et al., 2013; Drabińska et al., 2019; Rees, Burklund, et al., 2018; Schulz & Dickschat, 2007; Sethi et al., 2013). In light of these compounds largely arising from upstream metabolic processes, VOCs are representative of metabolic phenotype and offer valuable insight into the cellular state. Recent studies have demonstrated that differences exist in the VOC profiles between susceptible and resistant bacterial strains (Drabińska et al., 2022; Rees, Nasir, et al., 2018; Smart et al., 2019).

In the current study, we analysed the headspace of AmpC‐producing and nonproducing *E. coli* by means of thermal desorption‐gas chromatography‐mass spectrometry (TD‐GC‐MS) for the purposes of assessing microbial volatile metabolites. Using a metabolomics‐based approach, we sought to examine the differential volatile profiles produced in vitro by ampicillin‐susceptible and ‐resistant *E. coli* and to assess the influence of drug stress on these bacteria.

## METHODS

### pET16b plasmid transformation

Fifty microlitres of competent *E. coli* BL21 (DE3) cells (New England BioLabs) were combined with 5 ng of pET16b plasmid DNA (Addgene) and incubated on ice for 30 min. The pET16b plasmid contained the *ampR* gene and promoter for inducible expression of the *E*. coli chromosomal *ampC* gene. Contents were subjected to heat shock for 10 s at 42°C followed by a 5 min incubation on ice. Nine hundred and fifty microlitres of super optimal broth with catabolite repression media were added, and the transformed cells were incubated at 37°C with 180 rpm shaking for 1 h. The resulting culture was spread onto Luria‐Bertani (LB) agar plates containing 50 μg ml^−1^ ampicillin sodium (Formedium) and incubated overnight at 37°C for plasmid uptake selection.

### Bacterial cultures

Two *E. coli* nonpathogenic laboratory strains, BL21 (DE3) and pET16b plasmid‐transformed BL21 (DE3) (further referred to as BL21‐AmpC), were subcultured on LB agar plates and incubated at 37°C for 24 h. Single colonies of each strain were removed from plates and inoculated in 5 ml LB broth overnight at 37°C with 180 rpm shaking. Nitrocefin discs (Sigma‐Aldrich) were utilized to confirm β‐lactamase production in the plasmid‐transformed strain and absence in the nontransformed strain. The broth microdilution method was implemented for the determination of the ampicillin minimum inhibitory concentration (MIC) (The European Committee on Antimicrobial Susceptibility Testing, 2021). Each strain was assayed in triplicate, and the method was performed three times across three different days. Results were interpreted using the 2021 European Committee on Antimicrobial Susceptibility Testing (EUCAST) breakpoint criteria (The European Committee on Antimicrobial Susceptibility Testing, 2021).

### Headspace sampling from cultures

Overnight cultures of the two *E. coli* strains were standardized to 0.1 OD_600_ with LB broth (~1 × 10^8^ colony forming unit ml^−1^). One hundred microlitres of culture were inoculated in 20 ml headspace vials containing 1 ml supplemented LB broth. Three set‐ups were prepared for each strain: drug‐free (BL21 *n* = 7, BL21‐AmpC *n* = 7), low concentration ampicillin (2 μg ml^−1^; BL21 *n* = 10, BL21‐AmpC *n* = 10) and high concentration ampicillin (10 μg ml^−1^; BL21 *n* = 7, BL21‐AmpC *n* = 7). Noninoculated media controls were prepared for comparative purposes and background subtraction. Inert‐coated stainless steel HiSorb™ probes (Markes International) comprising a polydimethylsiloxane sorbent were inserted into the headspace. Headspace was passively collected over a 6 h incubation period at 37°C with 180 rpm shaking after which the probes were removed and inserted into stainless steel tubes for analysis.

### TD‐GC‐MS analysis

Samples were dry purged with N_2_ at 50 ml min^−1^ for 4 min to remove water residue and analysed by TD‐GC‐MS. A gaseous internal standard (1 ppmV *p*‐bromofluorobenzene in N_2_; Thames Restek) was spiked onto each sample prior to desorption. VOCs were thermally desorbed at 280°C for 5 min (TD100; Markes International) and then transferred with split injection (1:10) to a cryofocusing trap maintained at 0°C, which was subsequently flash heated to 280°C for 2 min. VOC separation was performed on an Agilent 7890B GC (Agilent Technologies) using an Agilent DB‐5ms column (30 m × 0.25 mm × 0.25 μm) with constant helium flow (1 ml min^−1^). The GC column oven was set to a linear temperature ramp programme with an initial temperature of 30°C, increasing at 7.5°C min^−1^ to 250°C (29.33 min total GC cycle time). After GC separation, VOCs were transferred to an Agilent 7010 MS to obtain mass spectra. The MS utilized an electron ionization (EI+) source set to 70 eV and 150°C, and a triple quadrupole mass analyser in full scan mode across a range of 40–300 *m/z* with an acquisition rate of 5 Hz.

### Data analysis

Spectral deconvolution was performed using MassHunter Quantitative Analysis software (Agilent Technologies) with a retention window size factor of 100 and delta *m/z* tolerance of 0.3 AMU left/0.7 AMU right. Detected peaks were aligned using a retention time window of ±0.1 min, and tentative peak identifications were performed by comparing mass spectra against the National Institute of Standards and Technology (NIST) mass spectral library (version 2014) (Wallace, 2014). Only compounds with a mass spectral match factor ≥70% were selected, with annotations listed as the compound name of the top match score. Integrated peak areas were normalized against the internal standard, log_10_ transformed and auto‐scaled. Manual background subtraction was undertaken for each peak by subtracting peak areas found in the media from those of the experimental spectra. Only those compounds which displayed positive peak areas after this subtraction in at least all bar one replicates were considered in the analysis. The ‘all bar one’ criterion was applied to ensure that any biological variation present within our small sample size did not skew results during this early exploratory stage. In the event of the absence of a peak, a nominal value of 1/5 of the minimum positive value was assigned for each variable. Peaks resulting from suspected environmental contaminants and artefacts were removed from the analysis, for example siloxanes and phthalate‐derived compounds. The retention index of each peak was calculated according to the IUPAC's definition of the temperature programmed Kovat's index equation (IUPAC, 1997). Calculated retention indices were compared against those in the literature using the NIST database. Compound identifications with retention indices deviating by more than 5% from the literature were rejected. Multivariate statistical analyses utilizing the unsupervised machine‐learning methods principal component analysis (PCA) and hierarchal clustering analysis (HCA) were performed using R v4.1.2 (R Core Team, 2021). Univariate statistical analysis was performed by Student's *t* tests (*α* = 0.05), and volcano plots were constructed in R to assess the differential expression of features.

## RESULTS

### MIC determination

The BL21‐AmpC strain demonstrated growth in ampicillin concentrations up to and including 128 μg ml^−1^, confirming the acquisition of ampicillin resistance. The MIC of the non‐AmpC‐producing strain (BL21) was determined to be 4 μg ml^−1^, classifying the strain as susceptible based on EUCAST breakpoint values (The European Committee on Antimicrobial Susceptibility Testing, 2021). The results from this assay were used to select the experimental ampicillin concentrations for the VOC analysis of ampicillin‐challenged bacteria. A low‐level ampicillin concentration of 2 μg ml^−1^ was selected, whereas 10 μg ml^−1^ was chosen for high concentration analysis.

### Core volatiles of untreated *E*. *coli* strains

The core volatilome was defined as the set of VOCs produced by both strains and included 42 compounds; an additional 14 compounds were detected in the susceptible strain. The pan‐volatilome was defined as the entire set of 66 volatile metabolites. Univariate analysis using Student's *t* test demonstrated that 31 compounds differed in abundance (*p* ≤ 0.05) between untreated susceptible and resistant strains. Five compounds were significantly increased (*p* ≤ 0.05) in the AmpC‐producing strain compared to the susceptible strain, identified as acetoin (retention time [RT] 3.563 min), 3‐methyl‐1‐butanol (RT 3.933 min), an unknown alkane (RT 14.552 min), indole (RT 14.902 min) and an unknown benzene derivative (RT 22.082 min). The detected compounds are detailed in Table 1. Annotations were compared to the published microbial VOC database (mVOC 2.0) (Lemfack et al., 2018).

**TABLE 1:** Core VOCs detected for untreated ampicillin‐susceptible and ‐resistant strains of *Escherichia coli* BL21

VOC	CAS			RT (min)	Calculated RI	RI NIST	Normalized peak area				*p*	Found in mVOC 2.0
		*m/z*					BL21		BL21‐AmpC			
		Quant	Qual				Mean	SD	Mean	SD		
Dimethyl sulfide	75‐18‐3	62.0	47.0, 45.0	2.244	507	516	260.25	120.27	54.54	22.87	<0.005	Y
Unknown 1 (2‐ketone)	51410‐11‐8	43.0	–	2.542	580	—	266.69	225.94	215.86	211.33	ns	–
Acetoin	513‐86‐0	45.0	88.0	3.563	704	706	26.32	9.31	36.79	10.04	ns	Y
3‐Methyl‐1‐butanol	123‐51‐3	55.0	70.0	3.933	729	727	846.09	141.02	1420.57	188.86	<0.005	Y
Dimethyl disulfide	624‐92‐0	93.9	–	4.080	739	748	1374.12	708.00	1588.52	671.16	ns	Y
2‐Methylthiazole	3581‐87‐1	98.9	57.7, 57.0	5.045	803	832	2.24	1.25	–	–	<0.001	N
3‐Methylbutanoic acid	503‐74‐2	60.0	87.0	5.579	830	835	4.92	4.95	5.31	5.52	ns	Y*
Unknown 2 (alcohol)	–	98.0	97.0, 53.0	5.927	848	–	2.85	2.18	–	–	<0.005	–
1‐Hexanol	111‐27‐3	56.0	69.0, 55.0	6.249	865	869	17.18	3.88	19.62	3.21	ns	Y
Unknown 3 (benzene derivative)	–	132.9	150.9, 134.9	6.775	892	–	191.38	120.00	190.35	108.15	ns	‐
2,5‐Dimethylpyrazine	123‐32‐0	80.9	–	7.118	909	911	33.32	17.02	28.45	13.82	ns	Y
Dimethyl trisulfide	3658‐80‐8	125.8	78.9	8.377	969	969	429.99	217.06	375.97	164.29	ns	Y
Hexanoic acid	142‐62‐1	60.0	73.0	8.457	973	974	114.82	71.74	157.58	173.38	ns	Y*
Phenol	108‐95‐2	93.9	66.0	8.494	975	976	116.80	43.26	84.71	49.26	ns	Y
2‐Ethyl‐1‐hexanol	104‐76‐7	57.0	70.0, 55.0	9.580	1026	1025	22.41	9.38	18.78	16.70	ns	Y
Benzyl alcohol	100‐51‐6	107.9	78.9, 77.0	9.704	1032	1035	79.95	15.21	83.82	20.59	ns	Y
1‐Ethenoxy‐2‐ethylhexane	103‐44‐6	71.0	–	9.772	1036	1038	2.95	0.92	3.60	1.62	ns	N
1‐Methyl‐2‐pyrrolidinone	872‐50‐4	98.9	98.0, 71.0	9.781	1036	1034	5.54	5.16	8.31	10.89	ns	N
Tetrahydro‐2H‐pyran‐2‐one	542‐28‐9	99.9	57.0, 56.0	10.039	1048	1010	1.02	0.60	–	–	<0.001	N
3‐Acetyl‐1H‐pyrroline	1072‐82‐8	93.9	108.9, 66.0	10.247	1058	1061	3.15	2.29	–	–	<0.005	N
Acetophenone	98‐86‐2	104.9	119.9, 77.0	10.388	1065	1065	121.25	65.87	149.80	120.26	ns	Y
3‐Ethyl‐2,5‐dimethylpyrazine	13360‐65‐1	134.9	135.9	10.579	1074	1079	9.31	4.28	–	–	<0.001	Y
2,6‐Diethylpyrazine	13067‐27‐1	135.9	134.9	10.767	1083	1085	2.21	1.60	–	–	<0.005	N
2‐Nonanone	821‐55‐6	57.7	71.0, 58.6	10.883	1089	1090	13.03	1.99	13.15	1.52	ns	Y
2‐Ethylhexanoic acid	149‐57‐5	88.0	73.0, 87.0	11.294	1109	1108	40.77	20.12	22.71	18.38	ns	N
Phenylethyl alcohol	60‐12‐8	91.0	91.9, 121.9	11.352	1112	1112	49.03	10.57	53.36	10.45	ns	Y
Unknown 4 (thiazole derivative)	–	111.9	112.9	11.512	1120	–	2.79	1.11	–	–	<0.001	–
Unknown 5 (benzene derivative)	–	117.1	296.8, 90.1	11.845	1136	–	11.86	2.94	5.55	2.54	<0.001	–
Unknown 6 (benzaldehyde derivative)	–	148.9	149.9, 131.9	12.208	1154	–	0.74	0.41	–	–	<0.001	–
1‐Nonanol	143‐08‐8	70.0	69.0, 56.0	12.515	1170	1173	3.28	0.95	3.63	0.58	ns	N
Naphthalene	91‐20‐3	127.9	–	12.890	1189	1188	5.65	3.72	–	–	< 0.005	N
Dodecane	112‐40‐3	71.0	57.0, 85.0	13.120	1200	1200	3.35	2.41	–	–	< 0.005	Y*
Decanal	112‐31‐2	82.0	55.0, 111.9	13.215	1205	1203	6.69	6.63	–	–	<0.05	Y
2,4‐Dimethylbenzaldehyde	15764‐16‐6	132.9	105.0, 77.0	13.458	1218	1217	3.66	0.73	2.63	1.49	ns	N
Unknown 7 (fatty acid methyl ester)	–	87.0	88.0, 71.0	13.535	1222	–	14.94	4.40	11.95	3.05	ns	–
3‐Phenylfuran	13679‐41‐9	143.9	114.9	13.578	1224	1224	6.68	2.50	–	–	<0.001	N
Benzothiazole	95‐16‐9	134.9	107.9, 73.0	13.662	1229	1228	13.98	9.32	–	–	<0.005	N
Unknown 8	–	58.6	102.9, 59.3	13.832	1237	–	0.75	0.65	–	–	< 0.01	–
3‐Butyl‐2,5‐dimethylpyrazine	40790‐29‐2	121.9	–	14.132	1253	1263	3.51	1.56	–	–	< 0.001	Y*
Unknown 9 (alkane)	–	71.0	85.0, 57.0	14.552	1275	–	0.92	0.82	3.81	4.95	ns	–
Indole	120‐72‐9	116.9	89.9, 88.9	14.902	1294	1293	60531.32	9898.92	78004.51	13472.80	< 0.05	Y
Unknown 10 (pyridine derivative)	–	119.9	134.9, 92.0	15.066	1303	–	8.92	2.36	5.63	2.71	< 0.05	–
Undecanal	112–44–7	67.0	82.0, 68.0	15.141	1307	1309	2.83	2.15	2.07	1.77	ns	N
Unknown 11 (ester)	–	144.9	102.9	15.652	1335	–	1.92	1.50	3.99	5.84	ns	–
4‐Methylquinoline	491‐35‐0	142.9	141.9, 114.9	16.495	1382	1384	2.73	2.44	1.62	1.17	ns	N
Tetradecane	629‐59‐4	57.0	71.0, 85.0	16.812	1400	1400	8.65	5.41	–	–	<0.001	Y
Unknown 12 (ketone)	–	82.0	109.0, 96.0	16.957	1409	–	5.07	2.99	–	–	<0.001	–
2,6‐Bis(1,1‐dimethylethyl)‐2,5‐cyclohexadiene‐1,4‐dione	719‐22‐2	176.9	134.9, 204.9	17.897	1464	1458	1.33	0.78	–	–	< 0.001	N
Unknown 13 (alkene)	–	109.9	96.0, 178.9	17.986	1469	–	9.13	1.92	7.86	0.88	ns	–
Unknown 14 (branched alkene)	–	55.0	–	18.049	1473	–	1.51	1.02	–	–	<0.005	–
2‐Tridecanone	593‐08‐8	57.7	71.0, 58.6	18.396	1494	1498	25.64	3.23	27.73	2.92	ns	Y
2,4‐Di‐tert‐butylphenol	96‐76‐4	190.9	–	18.593	1505	1518	175.87	103.49	62.08	20.83	< 0.05	Y*
Tridecanal	10486‐19‐8	82.0	96.0, 94.9	18.676	1510	1510	2.65	2.53	–	–	< 0.05	N
Dibenzofuran	132‐64‐9	167.9	138.9	18.887	1523	1521	1.51	1.13	1.48	2.05	ns	N
Unknown 15 (furan derivative)	–	101.9	173.8	19.089	1535	–	13.23	19.38	58.82	83.07	ns	–
Hexadecane	544‐76‐3	71.0	57.0, 85.0	20.110	1600	1600	2.61	1.79	–	–	< 0.005	Y*
(1‐Pentylhexyl)‐benzene	4537‐14‐8	91.0	160.9	20.546	1629	1620	1.16	0.78	0.96	0.78	ns	N
(1‐Ethylnonyl)‐benzene	4536‐87‐2	91.0	118.9	21.100	1667	1656	0.91	0.70	–	–	< 0.005	N
2‐Pentadecanone	2345‐28‐0	57.7	71.0, 58.6	21.582	1650	1697	38.77	6.17	36.93	6.35	ns	Y
Unknown 16 (branched alkane)	–	71.0	57.0, 85.0	21.632	1703	–	3.51	1.79	3.51	1.57	ns	–
(1‐Methyldecyl)‐benzene	4536‐88‐3	104.9	220.9, 91.0	21.684	1706	1692	1.59	1.00	–	–	< 0.001	‐
Unknown 17 (benzene derivative)	–	147.9	162.9, 91.0	22.082	1733	–	24.09	19.04	46.19	17.07	< 0.05	–
Octadecane	593‐45‐3	71.0	85.0, 57.0	23.078	1800	1800	1.87	1.29	–	–	< 0.005	N
2‐Heptadecanone	2922‐51‐2	71.0	57.7, 85.0	24.453	1893	1899	20.92	22.76	8.64	1.69	ns	N
5‐Dodecyldihydro‐2(3H)‐furanone	730‐46‐1	85.0	83.0	27.227	2080	2104	28.47	7.37	23.60	13.41	ns	N
Unknown 18 (alcohol)	–	71.0	85.0, 57.0	27.641	2107	–	63.33	53.50	54.77	26.81	ns	–

*Note*: ns, not significant (*p* > 0.05).

Abbreviations: CAS, chemical abstracts service number; NIST, National Institute of Standards and Technology; RI, retention index; RT, retention time; VOC, volatile organic compound.

^a^ Listed in mVOC 2.0 but not for *E. coli*.

### Effect of treatment with 10 μg ml^−1^ ampicillin on the volatilome

**FIGURE 1:**
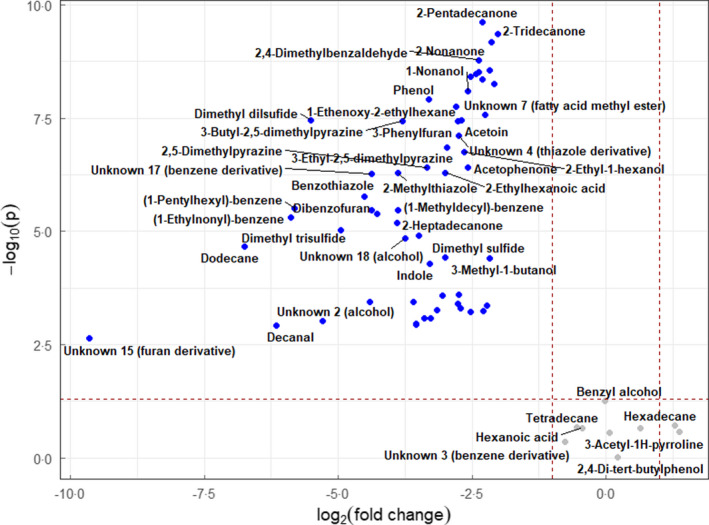
Volcano plot of volatile organic compounds detected in *Escherichia coli* BL21 strain in 10 μg ml^−1^ ampicillin‐supplemented media compared with drug‐free control with fold change threshold (x) 2 and *t* test threshold (y) 0.05. Blue (downregulated), grey (nonsignificant).

The volcano plot in Figure 1 depicts the differential VOC expression of the BL21 susceptible strain in the presence of 10 μg ml^−1^ ampicillin compared to the untreated condition. Of the 66 pan‐volatilome compounds, 57 were downregulated (fold change >2, *p* ≤ 0.05), with all but 14 compounds less than the limit of detection.

**FIGURE 2:**
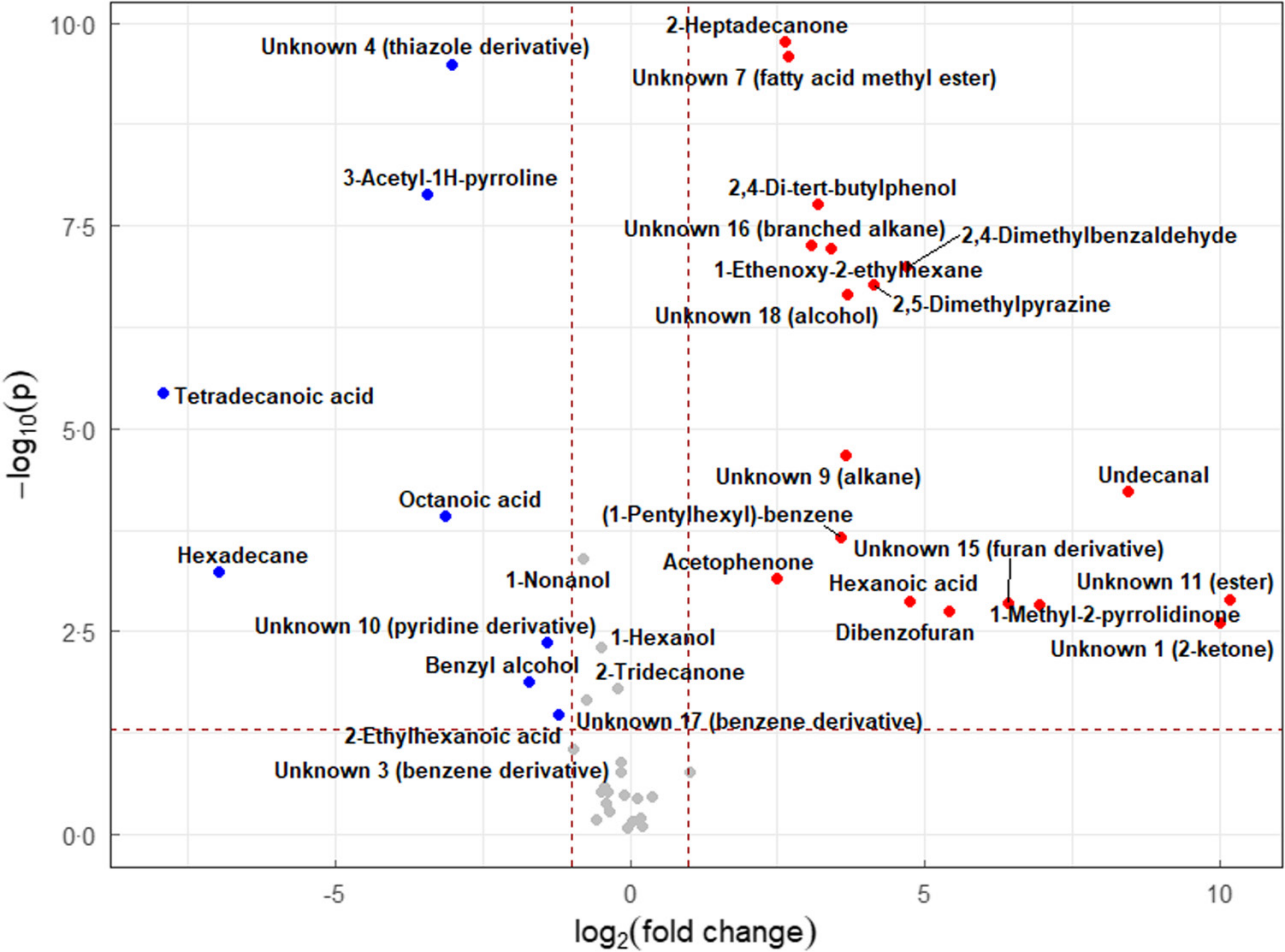
Volcano plot of volatile organic compounds detected in AmpC‐producing *Escherichia coli* BL21 strain in 10 μg ml^−1^ ampicillin‐supplemented media compared with drug‐free control with fold change threshold (x) 2 and *t* tests threshold (y) 0.05. Blue (downregulated), red (upregulated) and grey (nonsignificant).

The volcano plot in Figure 2 shows differentially expressed VOCs of the BL21‐AmpC strain in the presence and absence of 10 μg ml^−1^ ampicillin. A total of 26 altered VOCs (fold change >2, *p* ≤ 0.05) were observed when the AmpC‐producing strain was exposed to 10 μg ml^−1^ ampicillin compared to when the strain was grown in LB only. Compounds putatively identified as octanoic acid (RT 12.408 min) and tetradecanoic acid (RT 22.623 min) were detected in the headspace of the AmpC‐producing strain but were not previously observed in the pan‐volatilome. Two compounds which exhibited significantly higher levels (*p* ≤ 0.05) in the treated state (3‐acetyl‐1H‐pyrroline [RT 10.247 min] and an unknown thiazole derivative [RT 11.512 min]) were not previously observed in the volatilome of the untreated resistant strain, although they were detected in the untreated susceptible strain and formed part of the pan‐volatilome.

**FIGURE 3:**
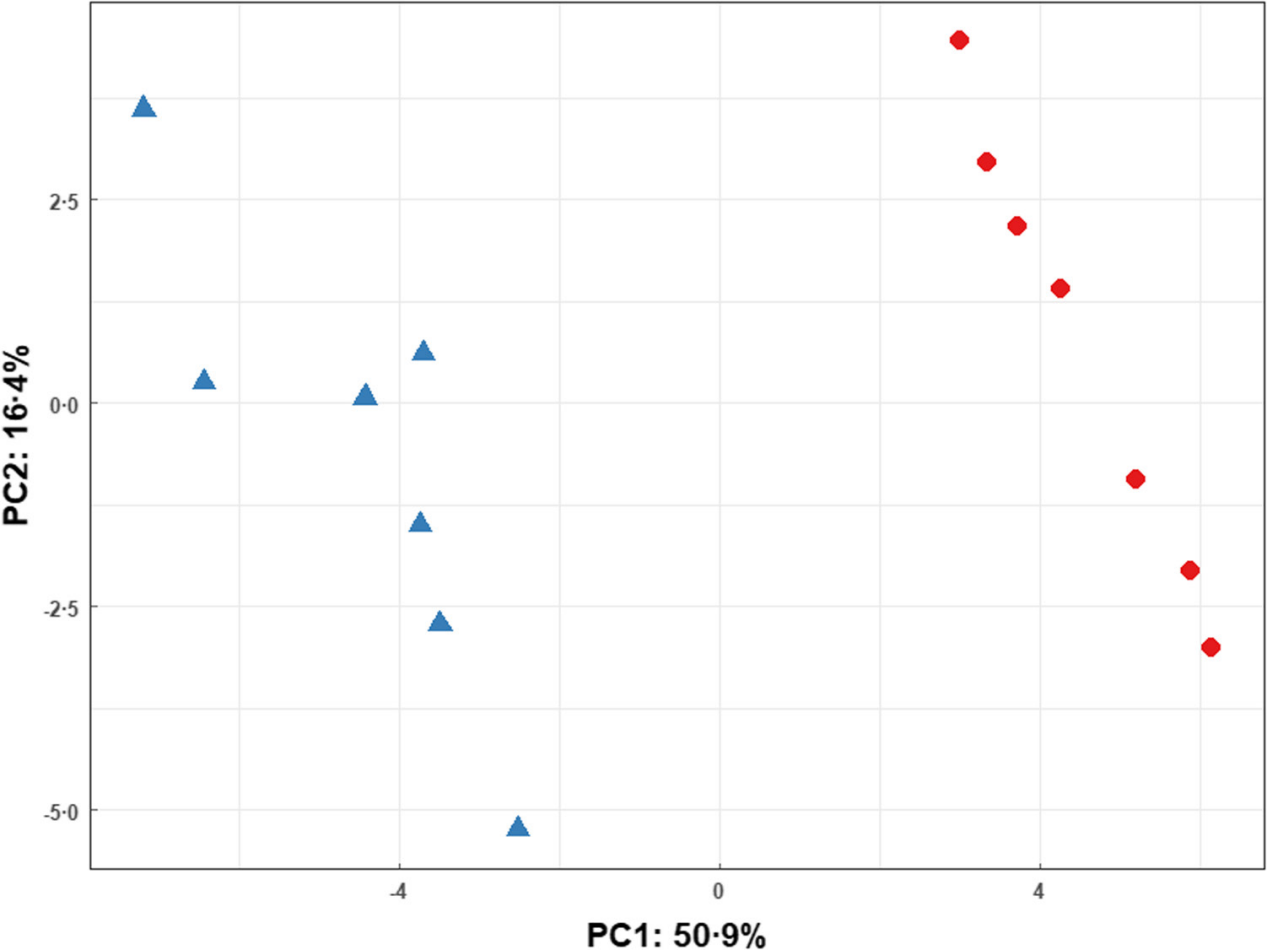
Principal component analysis scores plot comparing AmpC‐producing *Escherichia coli* BL21 strain in 10 μg ml^−1^ ampicillin‐supplemented media (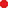) with drug‐free media (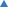).

Principal component analysis was implemented to determine whether the VOC profiles of the resistant strain in the presence and absence of 10 μg ml^−1^ ampicillin could be distinguished. Figure 3 demonstrates that the 10 μg ml^−1^ ampicillin‐stressed and unstressed BL21‐AmpC‐producing isolates separate across the first principal component (total explained variance = 50.9%).

### Treatment with 2 μg ml^−1^ ampicillin

**FIGURE 4:**
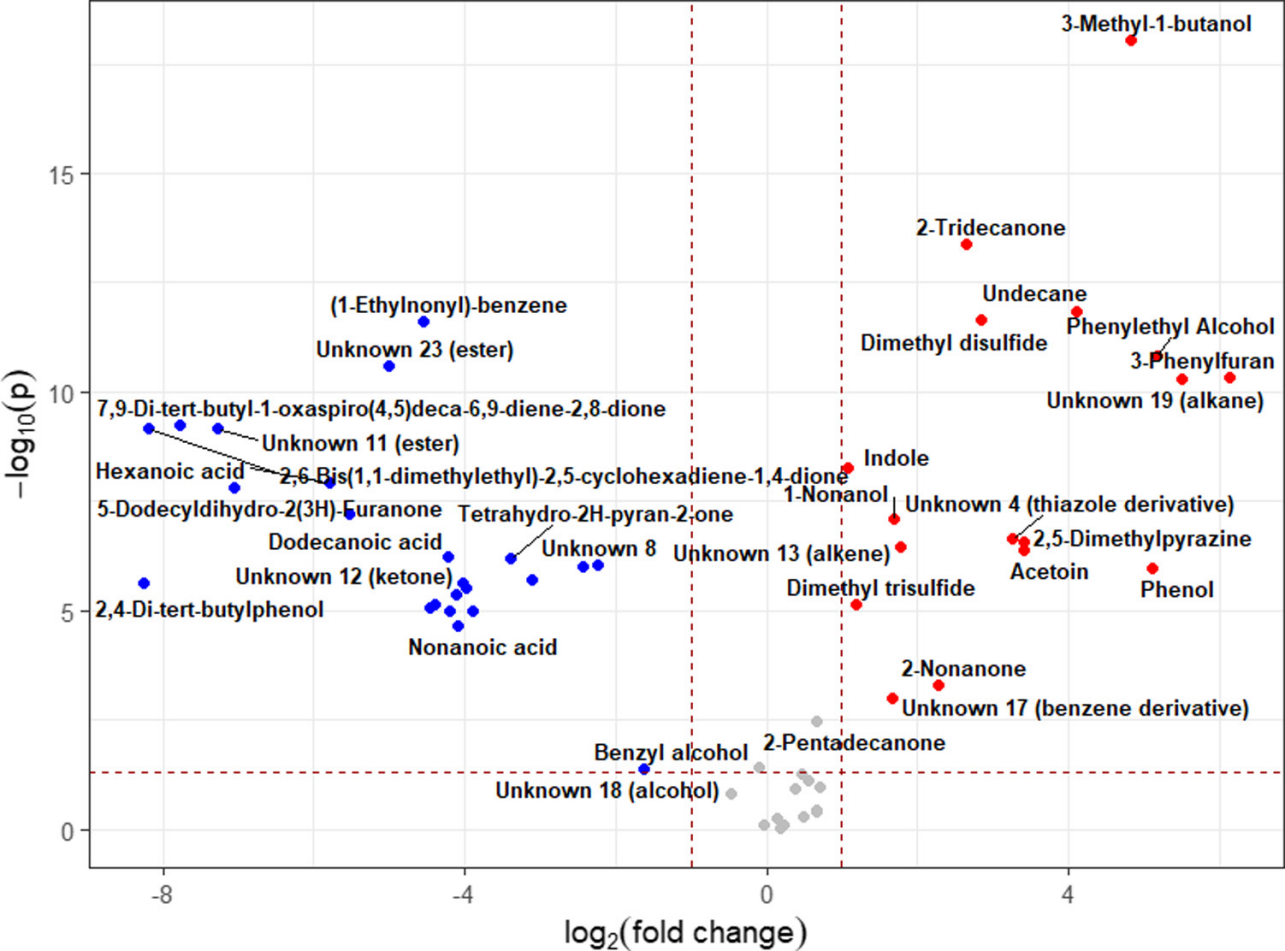
Volcano plot of volatile organic compounds detected in *Escherichia coli* BL21‐AmpC strain compared with *E. coli* BL21 strain in 2 μgml^−1^ ampicillin‐supplemented media with fold change threshold (x) 2 and *t* tests threshold (y) 0.05. Blue (downregulated), red (upregulated) and grey (nonsignificant).

The volcano plot (Figure 4) indicates 40 significantly altered VOCs (fold change >2, *p* ≤ 0.05) between the AmpC‐producing and nonproducing BL21 strains after incubation with 2 μg ml^−1^ ampicillin. Similar numbers of alcohols, heterocyclics, and aldehydes were detected between the two strains. The resistant strain showed a greater abundance of hydrocarbons (75%), whereas VOCs of the susceptible strain were enriched for ketones (64%), fatty acids (100%), esters (78%), and benzene‐derivatives (80%).

The heat map generated by HCA utilizing all detected compounds in both treated and untreated conditions (Figure S1) revealed distinct clustering patterns and differences between the four groups (BL21 untreated *n* = 7, BL21‐AmpC untreated *n* = 7, BL21 + 2 μg ml^−1^ ampicillin *n* = 10, BL21‐AmpC + 2 μg ml^−1^ ampicillin *n* = 10). The presence of drug stress in both strains correlated with higher abundances of pyrazine‐related compounds and lower abundances of several core volatiles such as phenol and 2‐pentadecanone. The BL21‐AmpC strain exhibited higher levels of other core volatiles including indole, 3‐methyl‐1‐butanol, dimethyl disulfide and dimethyl trisulfide under drug stress. Alkanes appeared enriched in the untreated BL21 group, whereas fatty acids were seen in greater abundance in the treated BL21 group.

**FIGURE 5:**
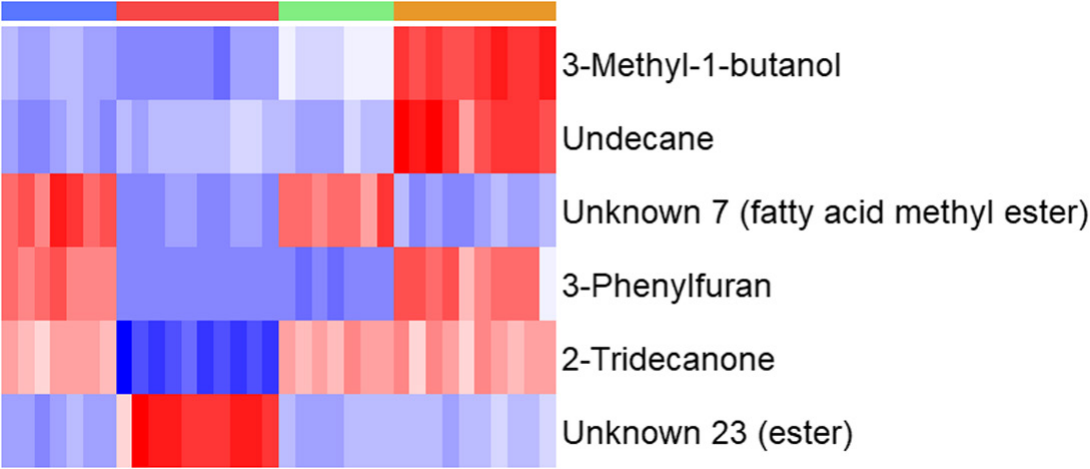
Heat map generated using hierarchical clustering analysis for the differentiation between AmpC‐producing and nonproducing *Escherichia coli* BL21 strains when treated with and without 2 μg ml^−1^ ampicillin using the top six identified compounds. Groups are represented in columns left to right by blue: BL21 (untreated), red: BL21 (2 μg ml^−1^ ampicillin), green: BL21‐AmpC (untreated) and orange: BL21‐AmpC (2 μg ml^−1^ ampicillin). Coloured cells correspond to compound peak intensities, with relative content for a given compound shown in red or blue to signify high and low values, respectively.

The top six discriminatory compounds identified by HCA were putatively identified as an unknown ester (RT 23.389 min), 3‐methyl‐1‐butanol, undecane (RT 11.112 min), an unknown fatty acid methyl ester (RT 13.535 min), 2‐tridecanone (RT 18.396 min), and 3‐phenylfuran (RT 13.578 min). HCA exclusively employing these six compounds (Figure 5) demonstrated clear differences between the groups.

**FIGURE 6:**
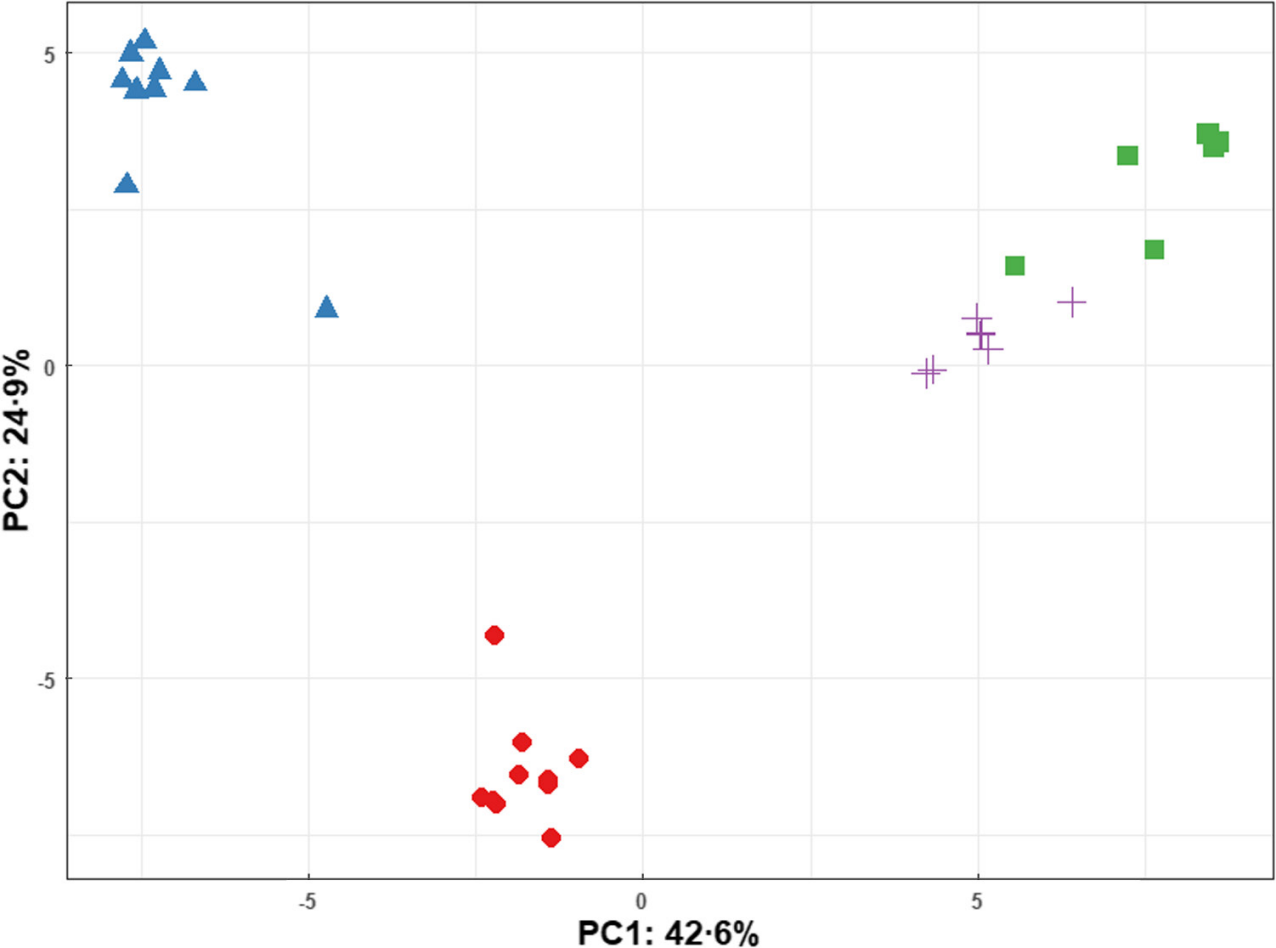
Principal component analysis scores plot comparing *Escherichia coli* BL21 and BL21‐AmpC strains in the presence and absence of 2 μg ml^−1^ ampicillin‐supplemented media. 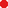 BL21 (2 μg ml^−1^ ampicillin), + BL21 (untreated), 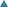 BL21‐AmpC (2 μg ml^−1^ ampicillin), 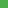 BL21‐AmpC (untreated).

The PCA scores plot (Figure 6) of the 2 μg ml^−1^ ampicillin‐treated and untreated strains showed excellent separation across the first and second PCs. The untreated strains did not exhibit substantial separation from each other.

We also sought to evaluate the effect of ampicillin concentration on the number of VOCs detected in the headspace of the two strains. In the untreated condition, the susceptible and resistant strains reported 66 and 42 compounds, respectively. Higher concentrations of ampicillin were correlated with lower numbers of detected VOCs in the susceptible strain (2 μg ml^−1^ *n* = 47, 10 μg ml^−1^ *n* = 14). However, while the number of detected VOCs decreased overall after subjecting the resistant strain to drug stress compared to the untreated condition, there was no significant difference in the detected number of VOCs between the 2 μg ml^−1^ (*n* = 32) and 10 μg ml^−1^ (*n* = 30) ampicillin set‐ups.

## DISCUSSION

We employed TD‐GC‐MS to assess the VOC profiles associated with AmpC‐producing and nonproducing *E. coli*, in order to evaluate any differences in the VOC profiles of ampicillin‐resistant and ‐susceptible bacteria under drug stress conditions. We identified several compounds which may be markers of AmpC production and by using machine‐learning algorithms demonstrated that the VOC profiles of ampicillin‐resistant and ‐susceptible bacteria are different. These findings indicate that bacteria with acquired resistance exhibit an altered metabolic phenotype compared to their susceptible counterpart and suggest that VOC profiles may be utilized to differentiate between resistant and susceptible strains of bacteria.

In the absence of drug stress, differences between the volatilomes of the resistant and susceptible strains were observed. Most compounds were found to be present or significantly increased in the susceptible strain and absent or significantly decreased in the resistant strain. However, five compounds were found to be significantly higher in the resistant strain suggesting that the metabolism of this strain is not completely downregulated in comparison. This is indicative of a fundamental biological difference in the metabolisms between the two strains.

Similar to previous findings, many of the compounds which were found to be significantly different between the untreated strains were no longer significantly different after the addition of a sublethal dose of ampicillin (Smart et al., 2019). The acquisition of resistance is known to confer a metabolic burden (Martinez & Rojo, 2011; Millan & Maclean, 2019). Thus, the absence of selection pressures for resistance reduces the competitiveness of the organism harbouring the resistance determinants. This may explain the lower abundance of VOCs detected in the untreated resistant strain as well as the detection of compounds under drug stress which were previously observed only in the untreated susceptible strain.

Three compounds (indole, acetoin and 3‐methyl‐1‐butanol) were observed to be significantly more abundant in the ampicillin‐resistant strain compared to the susceptible strain both in the presence and in the absence of drug stress. As well as having a role in tryptophan biosynthesis, indole has been implicated as a signalling molecule in the bacterial stress response as well as in the regulation of biofilm production (Di Martino et al., 2003; Kuczynska‐Wisnik et al., 2010). Indole signalling has also been shown to induce persistence in *E. coli* exposed to antibiotics and increase AMR (Vega et al., 2012). Acetoin has been shown to have a regulatory effect on motility and biofilm formation in bacteria (Létoffé et al., 2014). It is produced through the conversion of pyruvate and has been observed to prevent intracellular pH decreasing excessively (Vivijs et al., 2014). Modifications to the amino acid‐derived starter units for fatty acid biosynthesis may yield branched chain compounds, of which branched chain aldehydes are intermediates. For example, the metabolism of leucine may give rise to 3‐methylbutanal. The subsequent reduction by alcohol dehydrogenase may lead to the production of methyl alcohols, for example 3‐methyl‐1‐butanol (Schulz & Dickschat, 2007). Owing to the fact that LB broth contains tryptone and thus a range of amino acids, it is plausible that the high levels of 3‐methyl‐1‐butanol in the resistant strain have arisen due to the metabolism of culture medium amino acids (Filipiak et al., 2013). Our study showed that the two strains had significantly altered levels of VOCs involved in fatty acid biosynthesis and metabolism. The increased prevalence of fatty acids, for example octanoic and tetradecanoic acid in the headspace of the AmpC‐producing strain under drug stress may be explained by the biosynthesis of fatty acids. Conversely, the presence of smaller chain fatty acids in the susceptible strain under drug stress is likely resultant of fatty acid metabolism via the β‐oxidation pathway (Schulz & Dickschat, 2007). A multitude of other compounds are also produced along this pathway including 1‐alcohols, alkanes and 2‐substituted compounds.

To the best of our knowledge, this is the first time that sorptive extraction coupled with GC‐MS has been employed for the analysis of headspace volatiles of AmpC‐producing *E. coli*. Our experimental design aimed to control for confounding biological factors implicated in clinical isolates such as genetic determinants by utilizing plasmid transformation for inducible ampicillin resistance in BL21 (DE3) cells. Differences in the volatile profiles between the resistant and the susceptible strain may thus be attributed to the expression of *ampC* rather than a complex interplay of multiple factors as may be seen in clinical isolates. Furthermore, the application of ampicillin at a lower concentration permitted direct comparison between the susceptible and resistant strains without the influence of widespread cell death. This ensured that the experimental methodology detected VOC changes associated with AMR rather than detecting alterations correlated with inhibited metabolism and bacterial death as was observed when the susceptible strain was treated with a high concentration of ampicillin. Nonetheless, experimental groups assessing both low and high antibiotic concentrations provide insight into the effect of antibiotic concentration on the VOC profiles of bacteria. VOC analysis of the bacterial headspace at both levels showed significant differences between the VOC profiles and demonstrated potential applicability for the measurement of antimicrobial susceptibility. This is supported by previous studies which have monitored the growth and susceptibility of resistant and susceptible strains using VOCs (Allardyce et al., 2006; Wiesner et al., 2014).

Our findings suggest that ampicillin‐resistant bacteria may be distinguished from their susceptible counterparts using as few as six compounds within a time frame of <7 h. The development of computational models based on VOC signatures for the detection of AMR shows considerable promise for use in routine clinical diagnostics with little sample preparation and technical expertise required. Given the small sample size, future studies should look to expand the number of strains evaluated and assess other means of ampicillin resistance such as plasmid AmpC or alternative AmpC‐derived enzymes (e.g. CMY‐2). In addition, consideration should be given to clinical isolates as well as biological matrices to ascertain whether the same volatile signatures may also be detected in these typically complex samples. Validation of identified biomarkers will facilitate the development of targeted and rapid detection methods, reducing the incubation and analysis time currently required and promoting the timely identification of AMR‐associated infections.

## FUNDING INFORMATION

This work was supported by the Biotechnology and Biological Sciences Research Council (grant numbers BB/T008725/1 and BB/M011208/1). WMA, TF and SJF are supported by the NIHR Manchester Biomedical Research Centre.

## CONFLICTS OF INTEREST

The authors declare that they have no conflict of interest.

## Supporting information


Figure S1
