# Supplementary figures and images for: Metabolic phenotyping of acquired ampicillin resistance using microbial volatiles from Escherichia coli cultures
Source: J Appl Microbiol. 2022 Aug 2;133(4):2445–56. doi: 10.1111/jam.15716 (PMC9804386; doi:10.1111/jam.15716)

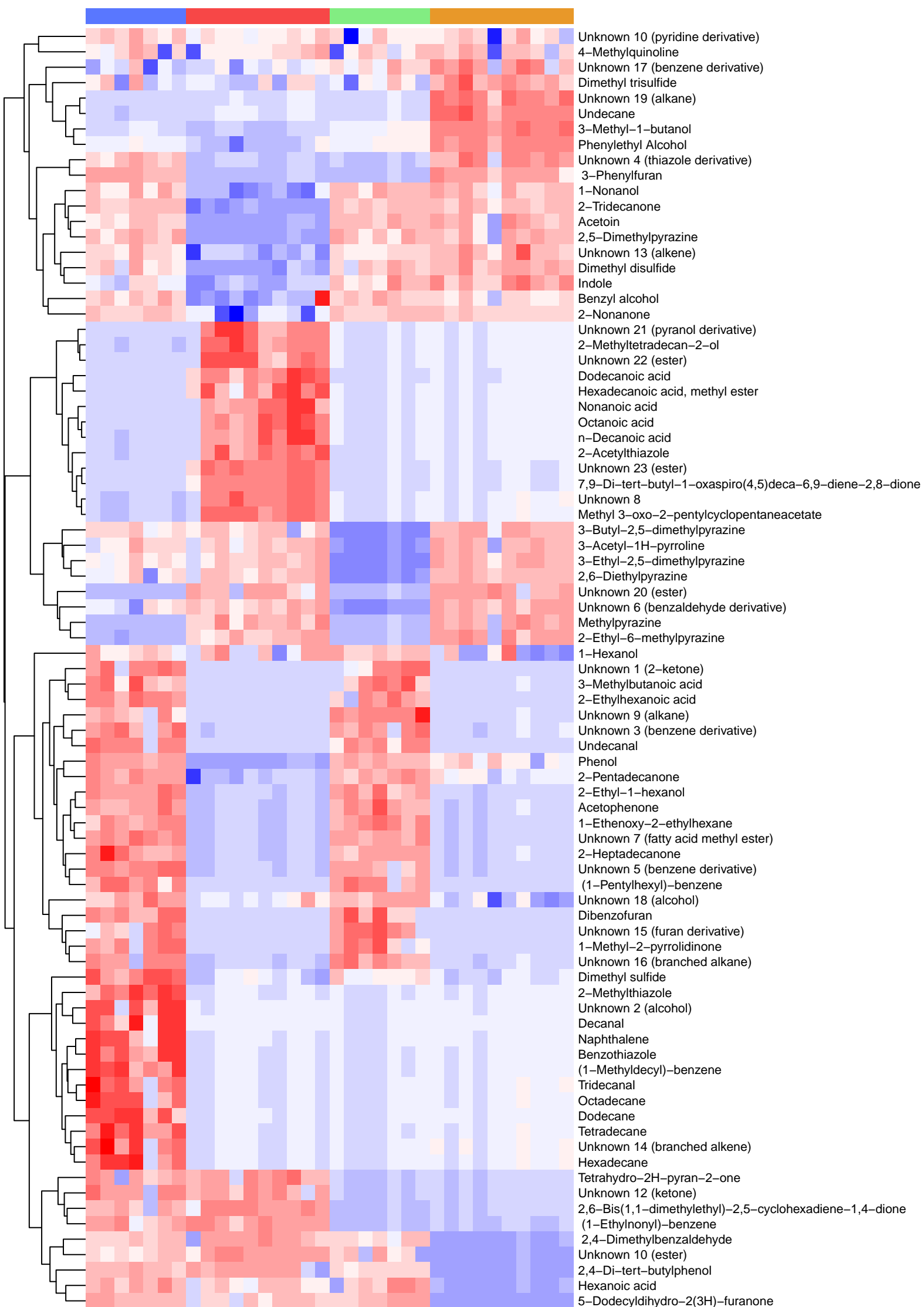

Supplement: Supplementary file 1 — Figure S1 [file JAM-133-2445-s001.pdf]
